# Individualized Strategies for Intraoperative Localization of Non-palpable Pulmonary Nodules in a Hybrid Operating Room

**DOI:** 10.3389/fsurg.2019.00032

**Published:** 2019-06-11

**Authors:** Osbert Qi Yao Leow, Yin-Kai Chao

**Affiliations:** Division of Thoracic Surgery, Chang Gung Memorial Hospital-Linko, Chang Gung University, Taoyuan, Taiwan

**Keywords:** small pulmonary nodules, image-guided thoracoscopic surgery, hybrid operating room, electromagnetic navigation bronchoscopy, microcoil

## Abstract

**Background:** Precise preoperative localization of small pulmonary nodules is a key prerequisite to their successful excision. With the advent of hybrid operating rooms (HORs), a patient-tailored approach encompassing simultaneous localization and removal of small pulmonary nodules has become feasible. In this study, we describe our individualized image-guided video assisted thoracoscopic surgery (iVATS) strategies implemented within a HOR environment. Specifically, localization was performed through different marking approaches (single- vs. double-marker) and access routes [percutaneous technique with Dyna-computed tomography (DynaCT) imaging vs. electromagnetic navigation bronchoscopy (ENB)].

**Methods:** Between April 2017 and November 2018, a total of 159 consecutive patients (harboring 174 pulmonary nodules) were treated with iVATS. The marking approach and access route were individually tailored according to lesion localization and its distance from the pleural surface. The efficacy and safety of our iVATS technique were determined through a retrospective review of clinical charts.

**Results:** All of the localization procedures were performed in a HOR by a single team of thoracic surgeons. The mean tumor size on preoperative CT was 8.28 mm (95% confidence interval [CI]: 7.6–8.96 mm), whereas their mean distance from the pleural surface was 9.44 mm (95% CI: 8.11–10.77 mm). Of the 174 tumors, 150 were localized through a percutaneous DynaCT-guided approach (single-marker: 139, dual-marker: 11), whereas localization in the remaining 24 was accomplished via the ENB-guided approach (single-marker: 4; dual-marker: 20). The mean localization time was 17.78 min (95% CI:16.17–19.39 min). The overall localization success rate was 95.9%. We failed to localize a total of seven nodules either because of technical complications (pneumothorax, *n* = 3; microcoil dislodgement; *n* = 1) or machine failure (*n* = 3). No operative deaths were observed, and the mean length of postoperative stay was 3.65 days (95% CI: 3.19–4.11 days).

**Conclusions:** The use of tailored marking approaches and access routes allowed us to individualize the iVATS procedure for small pulmonary nodules, ultimately promoting a more patient-centered workflow.

## Introduction

The number of small pulmonary nodules requiring assessment is continuously growing as a result of lung cancer screening using low-dose computed tomography (CT) (1). Nodules >5 mm with a solid component or enlarging lesions over time should generally prompt tissue sampling for diagnostic purposes (2). However, specimens obtained from percutaneous or bronchoscopic biopsies may frequently yield false-negative results, ultimately resulting in a low diagnostic accuracy. Although a more invasive diagnostic and therapeutic approach is advisable in this context, lesion excision through video-assisted thoracoscopic surgery (VATS) may be technically challenging (3). Precise preoperative lesion localization is a key prerequisite to their successful removal (4). Traditionally, small pulmonary nodules are excised through a two-step approach (lesion localization in a CT suite after which the patient is moved to an operating room) (5, 6).

With the advent of hybrid operating rooms (HORs), a patient-tailored approach encompassing simultaneous localization and removal of small pulmonary nodules has become feasible (7–9). Owing to the integration between high-quality imaging systems and surgical equipment, HORs have allowed localization and removal of small lung nodules to be simultaneously performed in a single session. In this context, image-guided video-assisted thoracoscopic surgery (iVATS) is emerging as a breakthrough technology to improve surgical outcomes and reduce complication rates (10). In this study, we describe our individualized iVATS strategies implemented within a HOR environment. Specifically, localization was performed through different marking approaches (single- vs. double-marker) and access routes [percutaneous technique with Dyna-computed tomography (DynaCT) imaging vs. electromagnetic navigation bronchoscopy (ENB), either with or without DynaCT].

## Methods

### Indications for Nodule Localization

Localization was performed in presence of ground-glass nodules (GGNs) or subpleural cavitary lesions. We also localized solid nodules that were either (1) subpleural and <10 mm in size or (2) deeply located in the lung parenchyma (distance between the lesion and the visceral pleural surface >10 mm).

### Localization Technique

**Figure 1:**
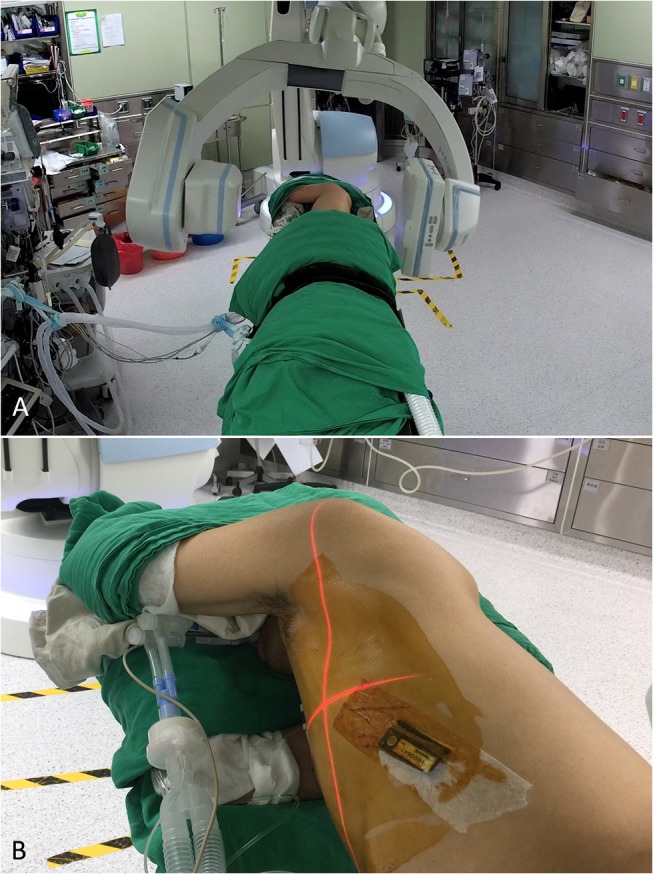
**(A)** With the patient lying in the lateral decubitus position, an initial scan for surgical planning was acquired using a standard 6s DynaCT body protocol during end-inspiration breath holding. **(B)** A laser-targeting cross was projected onto the patient's surface to visualize the needle entry point and angulation.

**Figure 2:**
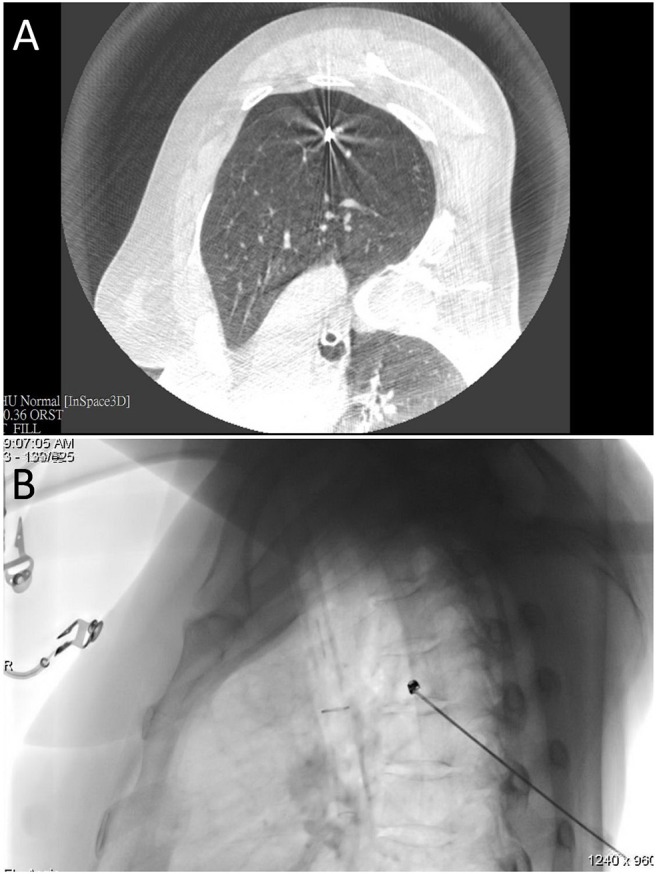
**(A)** An intraprocedural CT scan confirmed that the needle tip was properly positioned in the proximity of the nodule of interest. **(B)** Fluoroscopic view of microcoil insertion through a co-axial needle.

Percutaneous DynaCT-guided single- or dual-marker localization. The HOR in which all of the iVATS procedures were performed was equipped with a cone-beam CT apparatus (ARTIS zeego; Siemens Healthcare GmbH, Erlangen, Germany) and a Magnus surgical table (Maquet Medical Systems, Wayne, NJ, USA). Patients were placed in the lateral decubitus position after induction of general anesthesia. The cone-beam CT C-arm and the patient's chest were wrapped with sterile material. During end-inspiration breath holding, we acquired an initial scan for surgical planning using a standard 6s DynaCT body protocol (Figure 1A). We laid out the access path in the isotropic data set under the syngo Needle Guidance of a syngo X-Workplace (Siemens Healthcare GmbH). The needle path—which was outlined by marking the entry and target points—was subsequently projected onto the patient's skin with the use of a laser beam. A three-dimensional, laser-supported, fluoroscopic guidance was used to introduce an 18-gauge marker needle into the thorax during end-inspiration breath holding. The needle entry site and angulation were visualized by projected a laser-targeting cross onto the patient's surface (Figure 1B). Needle orientation and positioning were subsequently adjusted by pointing the planned, virtual needle path onto the live fluoroscopic image. A post-procedural cone-beam CT scan was acquired to confirm an appropriate needle positioning (Figure 2A). Superficial lesions were identified via a single-marker approach through injection (0.3 mL) of a dye, i.e., patent blue V (PBV 2.5%; Guerbet, Aulnay-sous-Bois, France) or indocyanine green (ICG) (11). Patent blue V dye was used in our initial cases and then we shifted to ICG marking. ICG fluorescence outshone the patent blue V dye as it remains detectable even in the pleura with anthracotic pigmentation; notably, such pleural modifications may hamper tumor detection with blue dye. Another point that merits consideration is that spillage of blue dye is difficult to remove. In contrast, extravasated ICG can be easily wiped off with gauze pads (11).

Lesions located deeply in the lung parenchyma were localized with a dual-marker approach. To this aim, the coaxial technique was used to place one Tornado® microcoil (model MWCE-35-8/5; Cook Inc., Bloomington, IN, USA) at the deep lesion margin (Figure 2B), with diluted dye being subsequently injected on the pleural surface surmounting the lesion. A post-procedural CT scan was performed to confirm that the microcoil was properly placed. The video of the procedure could be accessed at the following link (https://www.youtube.com/watch?v=2rPrvF3YnPo&t=7s).

#### Electromagnetic Navigation Bronchoscopy Single- or Dual-Marker Localization, Either With or Without DynaCT

**Figure 3:**
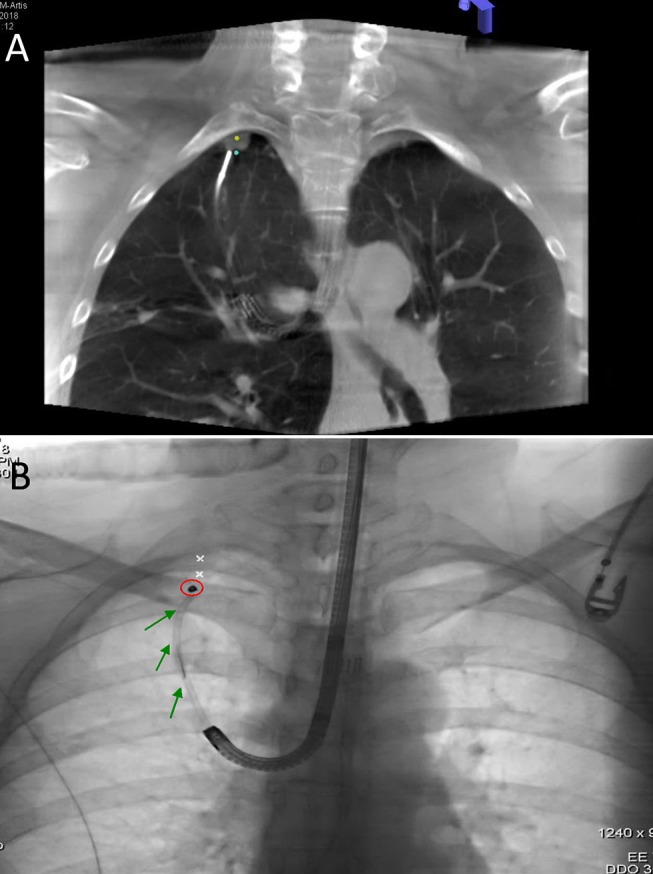
**(A)** An intraprocedural CT scan confirmed that LG was properly positioned at the deep margin (blue dot) of the nodule of interest (yellow dot). **(B)** Fluoroscopic view of microcoil (red circle) insertion through EWC (green arrow).

During the preoperative planning phase, a CT scan was acquired (1-mm slices). Mapping was performed with the super Dimension Electromagnetic Navigation Bronchoscopy (ENB) system (iLogic; Medtronic plc, Fridley, MN, USA). CT images were initially loaded onto the virtual bronchoscopy planning software, which subsequently reconstructed the axial, coronal, and sagittal virtual bronchoscopy views. Upon identification of different anatomical registration points (i.e., carina and all of the lobar orifices), we outlined the nodule of interest and mapped the path from the trachea to the target lesion. The navigation procedure was performed after placement of a single-lumen endotracheal tube. After a routine surveillance bronchoscopy, a locatable guide (LG)—which allows tracking the position and orientation within an extended working channel (EWC)—was inserted into the bronchoscope. Images acquired during bronchoscopy were matched with anatomical data obtained from CT scans, and the virtual planned path was thoroughly followed until LG reached the target nodule. An intraprocedural CT scan (Figure 3A) was subsequently acquired to investigate the positioning of the LG with respect to the nodule. Using the syngo iGuide Toolbox software package, the lesion of interest was selected on the CT scan and projected onto standard fluoroscopy. The LG was removed upon confirmation of its correct positioning on fluoroscopy, and a bronchoscopy needle was inserted into the EWC. When the needle was properly located, a 0.3 mL PBV or ICG dye solution was injected onto the pleural surface proximal to the lesion. When a dual-marker approach was required, a Tornado® microcoil (model MWCE-35-8/5; Cook Inc., Bloomington, IN, USA) was positioned at the proximal margin of the lesion upon dye injection (Figure 3B).

### Surgical Procedure Following Lesion Localization

A marker-guided VATS wedge resection was performed using a minimally invasive ICG fluorescence system (PINPOINT® Novadaq, Mississauga, ON, Canada) which included a 10-mm, 30° NIR thorcoscopic camera for the identification of the NIR tattoo, either with or without fluoroscopic assistance. The resected pulmonary specimen was subjected to frozen section examination. In general, lobectomy was used to treat patients with a confirmed diagnosis of primary lung cancer. Patients with peripheral lung malignancies of limited size (<2 cm) and adequate resection margins (either >2 cm or >tumor size) underwent sublobar resections.

### Statistical Analysis

The following data were extracted from a retrospective review of clinical charts: demographic characteristics of the study patients, results of cone-beam CT localization, surgical findings, and occurrence of complications. Continuous data are expressed as means with their 95% confidence intervals (CIs) or medians and interquartile ranges (IQRs). Categorical data are summarized as counts and percentages. All calculations were performed with the Statistical Package for the Social Sciences (SPSS), version 20 (IBM, Armonk, NY, USA).

## Results

Between April 2017 and November 2018, a total of 160 consecutive iVATS procedures were performed in 159 patients (one study participant received a bilateral iVATS procedure). The study sample consisted of 76 men and 83 women who harbored 174 pulmonary nodules (Table 1). The indications for surgery included suspected primary lung cancer in 139 tumors and suspected metastases in 35 tumors. The distribution of the localization techniques was as follows: single-marker DynaCT, *n* = 139; dual-marker DynaCT, *n* = 11; single-marker ENB, *n* = 4; and dual-marker ENB, *n* = 20 (Table 1). The distribution of the 174 pulmonary nodules was as follows: right upper lobe (RUL), *n* = 53; right middle lobe (RML), *n* = 19; right lower lobe (RLL), *n* = 43; left upper lobe (LUL), *n* = 44; and left lower lobe (LLL), *n* = 15 (Table 1). According to preoperative CT imaging, 102 lesions were subsolid and 72 were solid. The mean lesion size was 8.28 mm (95% CI: 7.6–8.96 mm). Overall, the mean distance between the closest pleural surface and the superficial border of the lesion was 9.44 mm (95% CI: 8.11–10.77 mm). Specifically, this distance was 9.26 mm (95% CI: 7.83–10.69 mm) in the single-marker DynaCT group; 4.46 mm (95% CI: 2.29–6.63 mm) in the dual-marker DynaCT group; 6 mm (95% CI: 0.7–11.3 mm) in the single-marker ENB group; and 14.14 mm (95% CI: 8.51–19.78 mm) in the dual-marker ENB group (Table 1).

**Table 1:** General characteristics of the study patients and the nodules of interest.

	**Total**	**Single-marker DynaCT**	**Dual-marker DynaCT**	**Single-marker ENB**	**Dual-marker ENB**
Number of patients	159	125	11	4	20
SEX
Male	76 (47.8%)	61 (48.8%)	3 (27.3%)	1 (25%)	11 (55%)
Female	83 (52.2%)	64 (51.2%)	8 (72.7%)	3 (75%)	9 (45%)
INDICATION FOR SURGERY
Suspected lung cancer	139 (79.9%)	109 (78.4%)	9 (81.8%)	3 (75%)	18 (90%)
Suspected metastases	35 (20.1%)	30 (21.6%)	2 (18.2%)	1 (25%)	2 (10%)
Number of nodules requiring localization	174	139	11	4	20
ANATOMICAL LOCATION
RUL	53 (30.5%)	40 (28.8%)	3 (27.2%)	1 (25%)	9 (45%)
RML	19 (10.9%)	15 (10.8%)	1 (9.1%)	0 (0%)	3 (15%)
RLL	43 (24.7%)	38 (27.3%)	2 (18.2%)	1 (25%)	2 (10%)
LUL	44 (25.3%)	34 (24.5%)	4 (36.4%)	1 (25%)	5 (25%)
LLL	15 (8.6%)	12 (8.6%)	1 (9.1%)	1 (25%)	1 (5%)
CT FINDINGS
Subsolid lesion	102 (58.6%)	83 (59.7%)	8 (72.7%)	2 (50%)	9 (45%)
Solid lesion	72 (41.4%)	56 (40.3%)	3 (27.3%)	2 (50%)	11 (55%)
TUMOR SIZE ON CT(mm)
Mean (95% CI)	8.28 (7.6–8.96)	7.97 (7.26–8.68)	6.3 (4.88–7.72)	11.5 (2–21)	10.87 (8.01–13.73)
Median (IQR)	7 (5–10)	6.5 (5–9.7)	6 (4.9–7.5)	10 (6.75–17.75)	9.5 (6–13)
TUMOR DEPTH ON CT(mm)
Mean (95% CI)	9.44 (8.11–10.77)	9.26 (7.83–10.69)	4.46 (2.29–6.63)	6 (0.7–11.3)	14.14 (8.51–19.78)
Median (IQR)	7 (3–14)	7 (3–14.5)	4 (1.8–8)	6 (2.75–9.25)	10.5 (5.6–20.5)

*CT, computed tomography; ENB, electromagnetic navigation bronchoscopy; RUL, right upper lobe; RLM, right middle lobe; RLL, right lower lobe; LUL, left upper lobe; LLL, left lower lobe; CI, confidence interval; IQR, interquartile range*.

The mean localization time was 17.78 min (95% CI:16.17–19.39 min). It was 15.10 min (95% CI:14.22–15.98 min) in single-marker DynaCT group; 19.72 min (95% CI:16.67–22.77 min) in dual-marker DynaCT group; 11 min (95% CI:6.50–15.50 min) in single-marker ENB group; and 36.65 min (95% CI:27.22–46.08 min).

The overall rate of successful targeting during localization was 95.9% (167/174). Specifically, it was 95.7% (133/139) in the single-marker DynaCT group, 100% (11/11) in the dual-marker DynaCT group, 100% (4/4) in the single-marker ENB group, and 95% (19/20) in the dual-marker ENB group (Table 2). The microcoil placement failed in one case which the coil was found to migrate retrogradely to a segmental bronchus and ultimately requiring bronchoscopic removal. There were three cases of unsuccessful targeting caused by puncture-induced pneumothorax, with the procedure being consequently suspended. Three additional failures were due to malfunctioning of the DynaCT robotic C-arm which were due to mechanical breakdown.

**Table 2:** Details of the localization procedures and surgical approaches.

	**Total**	**Single-marker DynaCT**	**Dual-marker DynaCT**	**Single-marker ENB**	**Dual-marker ENB**
Success targeting rate	167 (95.9%)	133 (95.7%)	11 (100%)	4 (100%)	19 (95%)
PROCEDURE COMPLICATION
Pneumothorax	17 (9.8%)	10 (7.2%)	5 (45.5%)	0 (0%)	2 (10%)
Hemorrhage	1 (0.6%)	1 (0.7%)	0 (0%)	0 (0%)	0 (0%)
SURGICAL APPROACH
Wedge resection	164 (94.3%)	135 (97.1%)	11 (100%)	3 (75%)	15 (75%)
Segmentectomy	6 (3.4%)	3 (2.2%)	0 (0%)	0 (0%)	3 (15%)
Lobectomy	4 (2.3%)	1 (0.7%)	0 (0%)	1 (25%)	2 (10%)
LOCALIZATION TIME(mins)
Mean (95% CI)	17.78 (16.17–19.39)	15.1 (14.22–15.98)	19.72 (16.67–22.77)	11(6.50–15.50)	36.65(27.22–46.08)
Median (IQR)	14 (12–19)	13 (12–17)	19 (17-22)	12 (8–13)	32 (17.25–55.25)
OPERATION TIME(mins)
Mean (95% CI)	88.70 (82.24–95.15)	89.05 (81.78–96.31)	68 (56.86–79.13)	138.75 (43.97–233.52)	87.65 (67.42–107.86)
Median (IQR)	79 (59.5–103)	79 (61–103)	74 (47–82)	163.5 (78.25–174.5)	77.5 (51.25–129.5)

*CT, computed tomography; ENB, electromagnetic navigation bronchoscopy; CI, confidence interval; IQR, interquartile range*.

Post-procedural DynaCT imaging revealed the presence of pneumothorax and hemothorax in 17 patients and one patient, respectively. Based on the results of frozen section examination, the implemented surgical approaches were as follows: wedge resection (*n* = 164), segmentectomy (*n* = 6), and lobectomy (*n* = 4). Conversion to thoracotomy was not required in any patient (Table 2).

The following post-operative complications were observed: prolonged air leaks (>5 days) in four patients, chylothorax in one patient, empyema in one patient, pneumonia in two patients, and stroke in one patient. Two patients (one with a prolonged air leak and one with an empyema) required re-operation (Table 3). The chest tube remained positioned for a mean of 1 day, whereas the mean length of hospital stay was 3.65 days (95% CI: 3.19–4.11 days; Table 3).

**Table 3:** Post-operative outcomes.

	**Total**	**Single-marker DynaCT**	**Dual-marker DynaCT**	**Single-marker ENB**	**Dual-marker ENB**
FINAL PATHOLOGICAL DIAGNOSIS
Primary lung cancer	77 (44.3%)	59 (42.4%)	6 (54.5%)	1 (25%)	11 (55%)
Metastatic lung cancer	26 (14.9%)	22 (15.8%)	1 (9.1%)	1 (25%)	2 (10%)
Benign lesion	71 (40.8%)	58 (41.8%)	4 (36.4%)	2 (50%)	7 (35%)
POST-OPERATIVE COMPLICATIONS
Prolonged air leak	4 (2.3%)	3 (2.2%)	0 (0%)	0 (0%)	1 (5%)
Chylothorax	1 (0.6%)	1 (0.7%)	0 (0%)	0 (0%)	0 (0%)
Empyema	1 (0.6%)	1 (0.7%)	0 (0%)	0 (0%)	0 (0%)
Pneumonia	2 (1.1%)	2 (1.4%)	0 (0%)	0 (0%)	0 (0%)
Stroke	1 (0.6%)	0 (0%)	0 (0%)	0 (0%)	1 (5%)
CHEST TUBE DURATION (DAYS)
Mean (95%CI)	1.93 (1.64–2.23)	1.94 (1.62–2.26)	1.09 (0.53–1.65)	1.5 (0.59–2.41)	2.4 (1.1–3.7)
Median (IQR)	1 (1–2)	2 (1–2)	1 (1–1)	1.5 (1–2)	1 (1–2.75)
POSTOPERATIVE LOS (DAYS)
Mean (95% CI)	3.65 (3.19–4.11)	3.6 (3.2–4)	2.9 (1.9–3.9)	3 (3–3)	4.45 (1.54–7.36)
Median (IQR)	3 (2–4)	3 (2–4)	2 (2–3)	3 (3–3)	3 (2–4)

*CT, computed tomography; ENB, electromagnetic navigation bronchoscopy; LOS, length of stay; CI, confidence interval; IQR, interquartile range*.

## Discussion

Compared with the traditional workflow, the implementation of HORs—which allow real-time imaging to be performed simultaneously to the operation—has resulted in a more patient-centered surgical approach (10, 12, 13). Recent years have witnessed an increased utilization of HORs in the field of thoracic surgery (9, 14). As far as patients with non-palpable small pulmonary nodules are concerned, iVATS performed in a HOR offers several advantages. First, patient relocation is not required—resulting in a lower likelihood of wire dislodgement. Second, intraprocedural complications can be promptly identified and managed in a timely fashion. Third, the use of general anesthesia during lesion localization markedly reduces emotional distress and physical discomfort. Finally, the integration of a CT suite within an operating theater improves the surgical workflow and minimizes the time at risk. Notwithstanding these benefits, an open issue that merits comment is the amount of radiation exposure associated with the use of iVATS. Accordingly, there have been reports suggesting that the radiation dose delivered in a HOR environment may be even higher than that of a traditional CT-guided localization—especially to the attending personnel (15, 16). This critical point deserves further scrutiny.

It is also noteworthy that the use of a percutaneous puncture to reach specific anatomical positions (e.g., lung apex, proximity of the diaphragm or major mediastinal organs, or locations behind chest wall structures like the scapula) may be risky and/or technically demanding. Under these circumstances, lesions should preferably be accessed through an endobronchial route. ENB—which offers the significant advantage of delivering no or minimal radiation doses to the patient—has been utilized from at least a decade for lung tumor biopsy and it has been recently adopted for pulmonary nodule localization as well (17–19). In ENB, an electromagnetic sensor technology is used in combination with virtual three-dimensional bronchial reconstruction of CT images (which can be paired to real bronchoscopic images). As soon as its tip reaches the lesion during the navigational phase, LG is retracted—ultimately leaving the EWC locked into the bronchoscope and allowing surgeons to deliver a steerable catheter for marker insertion.

Although ENB avoids radiation exposure, its use is not devoid of important caveats. One major limitation is that LG positioning with respect to the lesion is projected onto preoperative CT images (acquired during full inspiration) and should therefore be considered virtual (rather than acquired in real-time) (20). During bronchoscopy, the lung does not remain static but moves in accordance with respiration. Consequently, the location of the nodule of interest as observed during this procedure may differ significantly from that noticed on the CT scan acquired in the initial planning (during end-inspiration breath holding). In this regard, a previous study has shown that differences between virtual and real images may be as high as 2 cm, especially when nodules are located in the lower lobes (21). Under these circumstances, we reasoned that the combined use of DynaCT imaging and ENB would reduce potential discrepancies and increase the accuracy of lesion localization (22). To this aim, intra-procedural CT imaging was utilized as a real-time confirmatory tool for LG positioning (20, 22). This approach also allows identifying lesions that otherwise cannot be visualized fluoroscopically.

Some caveats of the current study merit consideration. First, the proposed dual marker approach for deeper lung tumor should be considered as preliminary owing to relatively small sample size. Second, our sample in the “dual-marker” approach was not just limited to patients with deep lesions but included some cases with superficial nodules as well. This was mainly performed because of the innovative nature of our technique, which was initially tested in more easily accessible lesions. Larger studies specifically focusing on the localization of deep nodules will be required to confirm the clinical utility of our dual-marking approach.

## Conclusions

The use of tailored marking approaches and access routes allowed us to individualize the iVATS procedure for small pulmonary nodules, ultimately promoting a more patient-centered workflow.

## Ethics Statement

The Institutional Review Board of the Chang Gung Memorial Hospital approved the study protocol (CGMH-IRB 201600671A3).

## Author Contributions

OL and Y-KC contributed to the conception and design of the study. Y-KC collected the study data, organized the dataset, and was in charge of follow-up. OL was in charge of statistical analysis and prepared the first draft of the manuscript. All authors have made a significant contribution to the manuscript, have read and approved the version submitted, and share responsibility for it.

### Conflict of Interest Statement

The authors declare that the research was conducted in the absence of any commercial or financial relationships that could be construed as a potential conflict of interest.
